# The Association between Home Healthcare and Burdensome Transitions at the End-of-Life in People with Dementia: A 12-Year Nationwide Population-Based Cohort Study

**DOI:** 10.3390/ijerph17249255

**Published:** 2020-12-10

**Authors:** Ping-Jen Chen, Chung-Han Ho, Jung-Yu Liao, Lisanne Smits, Chao A. Hsiung, Sang-Ju Yu, Kai-Ping Zhang, Irene Petersen, Elizabeth L. Sampson

**Affiliations:** 1 Centre for Dementia Palliative Care Research, Marie Curie Palliative Care Research Department, Division of Psychiatry, University College London, London W1T 7NF, UK; lisannesmits94@gmail.com (L.S.); e.sampson@ucl.ac.uk (E.L.S.); 2 Department of Family Medicine and Division of Geriatrics and Gerontology, Kaohsiung Medical University Hospital, Kaohsiung Medical University, Kaohsiung 807, Taiwan; 3 School of Medicine, Kaohsiung Medical University, Kaohsiung 807, Taiwan; 4 Department of Medical Research, Chi-Mei Medical Center, Tainan 710, Taiwan; ho.c.hank@gmail.com; 5 Department of Hospital and Health Care Administration, Chia Nan University of Pharmacy and Science, Tainan 717, Taiwan; 6 Institute of Population Health Sciences, National Health Research Institutes, Miaoli 350, Taiwan; jyliao@nhri.edu.tw (J.-Y.L.); hsiung@nhri.edu.tw (C.A.H.); 7 Faculty of Medicine, University of Amsterdam, 1105 AZ Amsterdam, The Netherlands; 8 Home Clinic Dulan, Taitung 959, Taiwan; bebe.yu@gmail.com; 9 Home Clinic Dulan, Taipei 106, Taiwan; falcony910425@gmail.com; 10 UCL Department of Primary Care and Population Sciences, University College London, London NW3 2PF, UK; i.petersen@ucl.ac.uk; 11 Barnet Enfield and Haringey Mental Health Trust Liaison Psychiatry Team, North Middlesex University Hospital, London N18 1QX, UK

**Keywords:** dementia, end-of-life, home healthcare, hospitalisation, national health program, palliative care, patient transfer

## Abstract

Background: For people with dementia, burdensome transitions may indicate poorer-quality end-of-life care. Little is known regarding the association between home healthcare (HHC) and these burdensome transitions. We aimed to investigate the impact of HHC on transitions and hospital/intensive care unit (ICU) utilisation nearing the end-of-life for people with dementia at a national level. Methods: A nested case-control analysis was applied in a retrospective cohort study using a nationwide electronic records database. We included people with new dementia diagnoses who died during 2002–2013 in whole population data from the universal healthcare system in Taiwan. Burdensome transitions were defined as multiple hospitalisations in the last 90 days (early transitions, ET) or any hospitalisation or emergency room visit in the last three days of life (late transitions, LT). People with (cases) and without (controls) burdensome transitions were matched on a ratio of 1:2. We performed conditional logistic regression with stratified analyses to estimate the adjusted odds ratio (OR) and 95% confidence interval (CI) of the risks of transitions. Results: Among 150,125 people with new dementia diagnoses, 61,399 died during follow-up, and 31.1% had burdensome transitions (50% were early and 50% late). People with ET had the highest frequency of admissions and longer stays in hospital/ICU during their last year of life, while people with LT had fewer hospital/ICU utilisation than people without end-of-life transitions. Receiving HHC was associated with an increased risk of ET (OR = 1.14, 95 % CI: 1.08–1.21) but a decreased risk of LT (OR = 0.89, 95 % CI 0.83–0.94). In the people receiving HHC, however, those who received longer duration (e.g., OR = 0.50, 95 % CI: 0.42–0.60, >365 versus ≤30 days) or more frequent HHC or HHC delivered closer to the time of death were associated with a remarkably lower risk of ET. Conclusions: HHC has differential effects on early and late transitions. Characteristics of HHC such as better continuity or interdisciplinary coordination may reduce the risk of transitions at the end-of-life. We need further studies to understand the longitudinal effects of HHC and its synergy with palliative care, as well as the key components of HHC that achieve better end-of-life outcomes.

## 1. Introduction

### 1.1. Background/Rationale

Dementia is currently one of the commonest causes of death in Western countries and will be one of the major and increasing global causes of serious health-related suffering [1,2,3]. People with dementia are at a high risk of experiencing hospitalisation or other care transitions [4], particularly towards the end-of-life [5,6,7]. Transitions and life-prolonging treatments near the end-of-life may be caused by the fragmentation of care, are burdensome for people with dementia, and may indicate poor-quality end-of-life care [8,9].

Burdensome end-of-life transitions for people with dementia, mainly investigated in the US and Europe [8,9,10], are defined as “early transitions (ET)” when there are multiple hospitalisations during the last 90 days of life or “late transitions (LT)” when occurring in the last three days of life. Gozalo et al. reported that burdensome transitions for Medicare nursing home residents with cognitive issues were associated with poorer-quality end-of-life care, including stays in intensive care units (ICU) in the last 30 days of life or late referrals to hospice care [9]. Using a linked administrative and clinical database in London, Leniz et al. found people with ET had a higher level of hospital use throughout the last year of life than people with LT [10]. However, there is a lack of population-based data of burdensome end-of-life transitions for people with dementia in Asia.

Previous observational studies have shown that palliative care was associated with fewer referrals to the emergency room (ER) and reduced use of life-sustaining treatments at the end-of-life for people with dementia [11,12,13]. However, access to palliative care for people with dementia in different countries is still very limited and is often implemented close to the time of death, because the end-of-life trajectory is difficult to predict [11,14,15]. Besides, evidence of the effectiveness of palliative care for people with dementia living outside nursing homes is still poor [16]. Strategies and interventions to reduce avoidable transition and acute healthcare utilisation at the end-of-life for people with dementia living in the community are therefore vital.

Home healthcare (HHC) is increasingly recognised as important in supporting people with dementia living in the community [17]. HHC mostly comprises services that rehabilitate after an illness or injury or help to manage chronic diseases and their complications [18]. The services may include some aspects of social care but primarily focus on medical services provided by healthcare professionals or staff, such as physicians, registered nurses, pharmacists, or qualified therapists. Various programs are included in the category of HHC, such as Medicare skilled home healthcare, home-based primary care, physician house calls, or hospital at home. These vary in terms of acuity, type of care provided, and the degree of physician involvement [18,19,20].

Previous studies that investigated HHC interventions for similar end-of-life outcomes in people with dementia were mostly in small samples or followed a short period before death [21,22]. Jennings et al. reported a longitudinal nurse practitioner dementia care programme in the Los Angeles community that enrolled 322 people and achieved a more than 90% rate of advance care planning (ACP) in decedents. Programme recipients experienced fewer ICU stays and recurrent hospitalisations, consistent with their preferences for less aggressive care [21]. Mitchell et al. found that people aged 65 years or older with advanced dementia who died within one year of receiving HHC (*n* = 290) had fewer end-of-life hospitalisations than those that died within one year of admission to nursing homes (*n* = 2730) [22]. HHC service models in Taiwan are similar to the mix of home-based primary care and skilled home healthcare in the US Medicare system and have been reimbursed by the Taiwan National Health Insurance (NHI) scheme since 1995. The impact of “routine” HHC on end-of-life transitions in people with dementia in a larger-scale study for a longer follow-up has not been well-investigated.

### 1.2. Objectives

Our population-based cohort study aimed to investigate the patterns and characteristics of HHC utilisation, burdensome transitions, and hospital/ICU use at the end-of-life for people with dementia following from the point of their dementia diagnosis. We examined the association between HHC and burdensome end-of-life transitions and the impact of characteristics of HHC on these transitions at a national level in Taiwan.

Our hypothesis was that more continuous or more interdisciplinary collaboration in HHC may be associated with a lower risk of burdensome transitions in people with dementia at the end-of-life.

## 2. Materials and Methods

### 2.1. Setting

The NHI in Taiwan, initiated in 1995, provides single-payer healthcare services and covers 99.9% of Taiwan’s legal residents [23]. Almost all healthcare providers have contracts with the NHI, and all kinds of healthcare services are unified, with corresponding fees and reimbursements by the NHI mainly based on a fee-for-service model. By law, every beneficiary pays the NHI premium according to their income. The premiums of those who have no salary (so-called “dependent” group), such as spouses or elderly people, are paid by a family member who has a salary. The contents of healthcare services are the same regardless of the premium paid, and 10 percent of the cost of each healthcare service is co-payed by the beneficiaries unless the beneficiary has a “catastrophic illness certificate” where co-payment is waived.

HHC in Taiwan covers nurses’ and physicians’ visits, drug injections, respiratory therapy, and laboratory tests (before 2015), with extended services provided by more disciplines added after 2015 [24]. HHC is provided by HHC teams affiliated to hospitals, by independent HHC organisations, or by community health centres from the public sector [25]. The eligibility criteria for HHC in Taiwan cover those people who have (1) a limited ability to self-care, e.g., needing help with more than half of their daily activities; (2) definite medical or nursing care needs assessed by both physicians and nurses at outpatients clinics, on hospital admission, or at home; and (3) chronic conditions requiring long-term nursing care or continual post-discharge healthcare needs [25,26]. HHC in this study did not include the home-based palliative care (HBPLC) program in Taiwan.

### 2.2. Data Source and Ethics

This is a nationwide cohort study using Taiwan’s National Health Insurance Research Database (NHIRD), which is a routinely collected, claim-based, and anonymised electronic record database, including the entire inpatient and ambulatory healthcare services between 2000 and 2013. In the NHIRD, information about any utilisation of NHI-reimbursed healthcare services or drug prescriptions for an individual is obtained by tracing specific administrative codes and is linked by an encrypted identification code. Disease diagnoses are based on the International Classification of Diseases, Ninth Revision, Clinical Modification (ICD-9-CM) codes before 2016. The study protocol was approved by the institutional review board of Chi-Mei Medical Center in Taiwan (Approval No. 10410-E01) and informed consent waived due to the anonymous nature of NHIRD analysis.

### 2.3. Cohort Definition

**Figure 1:**
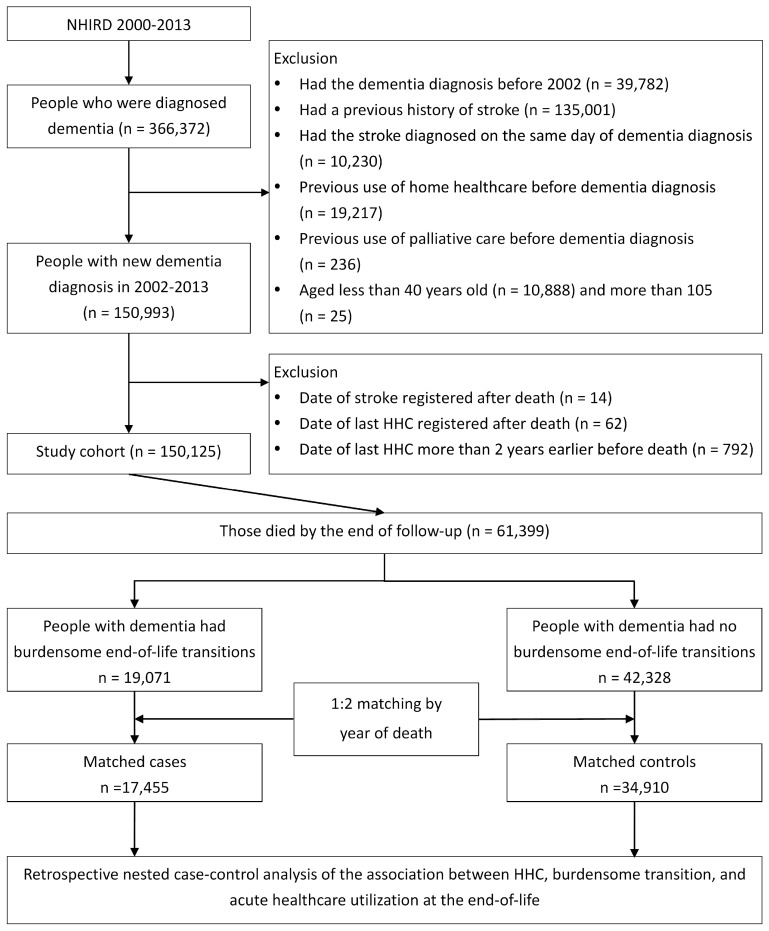
Schematic illustration of the study cohort and patient selection criteria. HHC = home healthcare and NHIRD = National Health Insurance Research Database.

The study cohort included people aged 40 years and older who had a new dementia diagnosis from the whole country from 2002 to 2013. Individuals with dementia were identified by the ICD-9-CM codes for dementia (Table A1 in the Appendix A), followed by at least one inpatient record of an ICD-9-CM dementia code or at least three outpatient records within 1 year. Cohort entry date was defined as the date on which the first diagnosis code of dementia appeared. We excluded people aged older than 105 years, individuals who had a previous history of dementia or stroke, had a stroke diagnosed on the same day of the dementia diagnosis, received HHC or palliative care before the cohort entry date, or received the last HHC in the follow-up more than 2 years before death. Those who had errors in the data registration, such as stroke, HHC, or palliative care recorded after death, were also excluded (Figure 1).

Next, people with dementia who died by the end of 2013 were selected as the study focus. Death was determined when a patient was coded as “in-hospital death” or “sudden death” at the ER or withdrew from the NHI programme without any other healthcare visits in the following 6 months, as is a common working criteria for the NHIRD [11].

### 2.4. Case Identification

A nested case-control analysis in cohort decedents was applied to minimise the bias of identifying end-of-life outcomes. We identified people with dementia who experienced end-of-life burdensome transitions before death, including ET or LT. ET was defined as three or more hospitalisations in the last 90 days of life, while LT was defined as any ER visit or hospitalisation in the last three days of life [9]. This pattern of transition was used as a binary variable in the analysis.

### 2.5. Exposure Measurements and Covariates

We examined all records of HHC received by people with dementia during the follow-up period before death (for the claim codes for HHC, see Table A2 in the Appendix A). Characteristics of HHC, including resource utilisation groups (RUG); total counts of services or services provided by different professionals; and time-related factors for services, such as the period from cohort entry, duration of HHC, and the period from last HHC to death, were presented and used in the subgroup analyses. HHC services by nurses can be classified into four levels according to RUG [26]. The first level RUG is intended for those who only require ordinary healthcare visits to the home, and levels 2–4 of RUG are aimed at people who require one, two, three, or more specialised care services, respectively. The predefined specialised care services include tracheostomy care; urinal indwelling catheterisation and/or catheter change; insertion and/or change of a nasogastric tube; bladder irrigation; wound care or debridement for stage 3 or 4 pressure injuries; intravenous fluid supply; colostomy, ileostomy, or urostomy care (e.g., an ileal conduit); or post-colon resection care. The reimbursement for physician visits is a fixed payment regardless of the RUG of HHC that patients receive [26].

Covariates included age, sex, socioeconomic status (personal income and urbanisation level of residence), comorbidities, and age-adjusted Charlson Comorbidity Index (aCCI) at baseline [27]. The aCCI scores were calculated with different comorbid conditions that were weighted and additional points added for age. Each decade over the age of 40 years was assigned a comorbidity score of 1 to risk and so on. Comorbidities and aCCI were defined based on information from at least one inpatient record or three outpatient records within 1 year before the cohort entry date and classified by ICD-9-CM codes (Table A1 in the Appendix A). Income was classified by insurance premiums, and urbanisation level was considered according to population density and medical resource [28].

### 2.6. Statistical Analysis

The mean and standard deviation (SD) or median and interquartile range was estimated for continuous variables. Discrete variables were described as the frequency with percentage. People with (cases) and without (controls) burdensome end-of-life transitions were matched on a ratio of 1:2 by year of death to diminish the influence of changes to policy or end-of-life care practice over time. Conditional logistic regression was then used to estimate the adjusted odds ratio (OR) and 95% confidence interval (CI) of risks of transitions between cases and controls. Stratified analyses for potential factors of interest were estimated and presented as forest plots. In addition, characteristics of HHC among people who received HHC were estimated using unconditional logistic regression analysis. All regression analyses were adjusted for age, gender, socioeconomic status, aCCI, and listed comorbidities. SAS 9.4 (SAS Institute, Cary, NC, USA) was used for all statistical analyses.

## 3. Results

### 3.1. Participants

**Figure A1:**
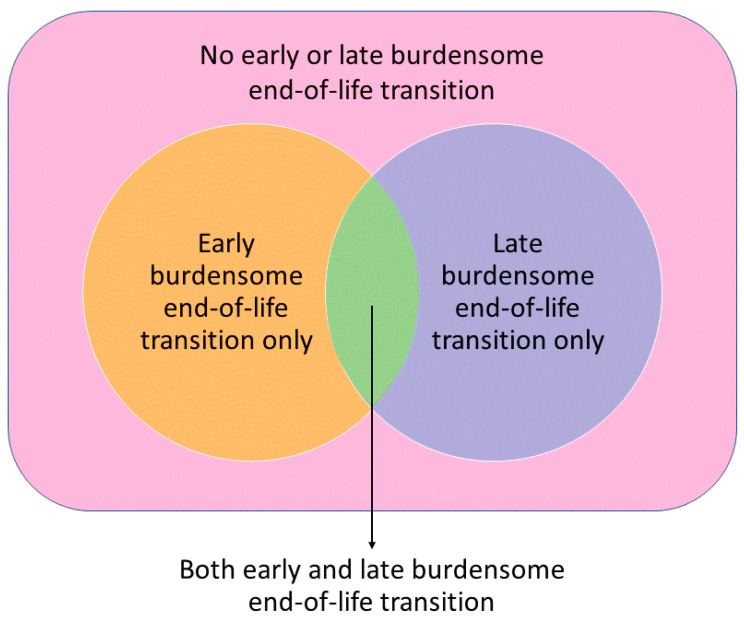
Illustration of people with dementia in different groups with or without burdensome end-of-life transition. In the regression model, the early burdensome transition represents those with early transition only and those with both early and late transition; the late burdensome transition represents those with late transition only.

Among 150,125 people with a new dementia diagnosis, a total of 61,399 decedents were identified. The mean age of decedents was 83.2 years at death, and the mean survival period was 1030.7 days after the diagnosis of dementia, 48.2% of whom were female (Table 1). The percentage of burdensome end-of-life transitions in the decedents was 31.1% (19,071), including 9533 with ET (15.5%) and 9538 with LT (15.5%). We further included 17,455 cases with burdensome end-of-life transitions, who were divided into different groups according to ET, LT, or both (Figure A1 in the Appendix A), and 34,910 controls for the nested case-control analysis.

### 3.2. Descriptive Data

Most characteristics at the time of dementia diagnosis were significantly different in proportions between cases and controls, and the group with ET had a much higher proportion of males (Table 1). For the characteristics at follow-up, the groups with ET had a shorter time from dementia diagnosis to death or to receiving the first HHC, shorter duration of HHC, and lower frequency of HHC compared to the group with LT only or controls.

**Figure 2:**
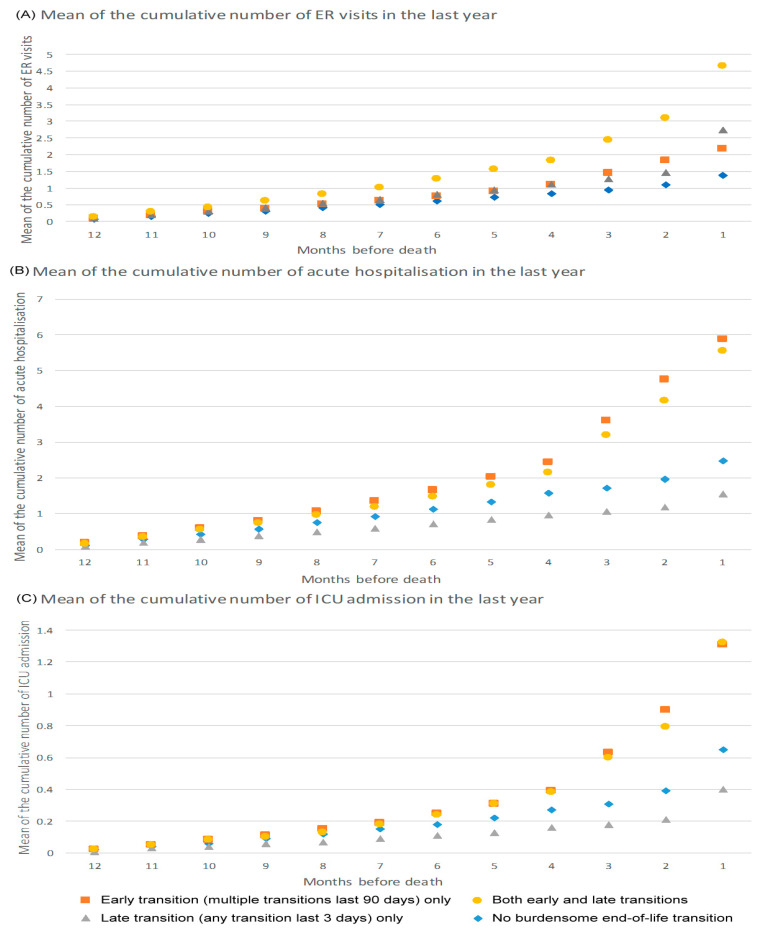
The utilisation of acute healthcare in the last year for people with dementia by type of burdensome end-of-life transition. ER = emergency room and ICU = intensive care unit.

Figure 2 presents the utilisation pattern of ER visits and hospital and ICU admissions in the last year of life for each group of cases and controls. The group with ET had more hospital/ICU admissions and longer hospital/ICU stays. The group with LT only had fewer hospital/ICU admissions than the control group during their last year.

### 3.3. Main Results

**Figure 3:**
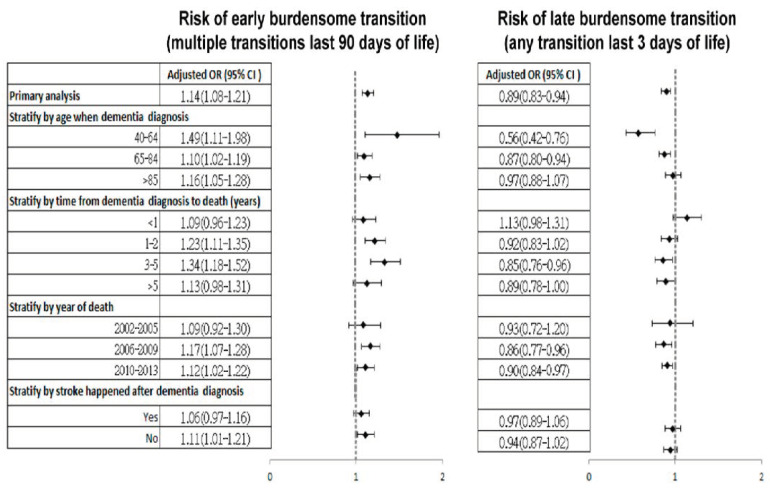
Risk of burdensome end-of-life transition in people with dementia who received home healthcare compared with those who had no home healthcare services (reference group). CI = confidence interval, HHC = home healthcare, ICU = intensive care unit, and OR = odds ratio. In the regression model, the early burdensome transition represents those with early transition only and those with both early and late transition; the late burdensome transition represents those with late transition only. All the analyses were adjusted for age, gender, socioeconomic status, age-adjusted Charlson Comorbidity Index, and comorbidities.

The risk of burdensome end-of-life transitions between those with and without HHC and the stratified group analysis are shown in Figure 3 (for the number of cases and controls, see Table A3 in the Appendix A). People who received HHC had a higher risk of ET than those without HHC (OR = 1.14, 95 % CI 1.08–1.21), whereas people with HHC had a lower risk of LT (OR = 0.89, 95 % CI 0.83–0.94). In the stratified group analysis, considering the age groups, survival period, or stroke event after dementia diagnosis and year of death, the risk of transitions between those with and without HHC is in keeping with the risk in the primary analysis. The only exception was in people whose survival period was less than one year after dementia diagnosis; those with HHC had a higher risk of LT than those without HHC (OR = 1.13, 95 % CI 0.98–1.31). A sensitivity analysis suggested similar results with the primary analysis after excluding people with missing values on urbanisation (Table A4 in the Appendix A).

Table 2 shows the influence by characteristics of HHC on the risk of burdensome end-of-life transitions in people who received HHC. The risk of transitions did not significantly differ between RUG levels or the period from dementia diagnosis to the first HHC. HHC that was more frequent (e.g., OR = 0.52, 95 % CI 0.45–0.61, >16 versus ≤4 times per year), of longer duration (e.g., OR = 0.50, 95 % CI 0.42–0.60, >365 versus ≤30 days), or delivered closer to the time of death (e.g., OR = 0.73, 95 % CI 0.62–0.84, >90 versus 0–15 days) was associated with a remarkably lower risk of ET. HHC that was of longer duration or provided by both physicians and nurses was associated with a lower risk of LT.

## 4. Discussion

We used a large database to conduct the first national-level cohort study on the impact of HHC on burdensome end-of-life transitions in people with dementia, following from dementia diagnosis to death. HHC, in general, is not specifically targeted at end-of-life care needs and, thus, is seldom investigated for its effect on end-of-life outcomes. Our findings have important implications for focusing interventions on those who experience ET and have the highest acute healthcare needs at the end of life. We need to understand further the differential effects of routine HHC on ET and LT and the specific components of HHC that reduced the risk of end-of-life transitions.

### 4.1. End-of-Life Transitions and Hospital/ICU Utilisations

Overall, in Taiwan, 31.1% of decedents with dementia experienced end-of-life transitions, a much higher proportion than the counterparts reported in US Medicare (19%) or Finish nursing home residents (9.9%), as well as in the UK (16%) [8,9,10]. The proportions of ET and LT among people with dementia were equal in our cohort, which is different to the LT-predominant pattern in the US and UK [9,10]. Previous studies found that younger age and male sex increase the risk of burdensome transitions, especially ET, in people with dementia [8,9,10]. Our findings on age and sex are in keeping with the existing literature. In addition, Leniz et al. reported that ET was associated with the presence of physical illness and depressed mood [10], which may explain the higher proportion of physical and mental comorbidities and higher aCCI score in cases with ET in our study.

A tendency towards aggressive healthcare at the end of life for people with dementia in Taiwan is associated with cultural norms of avoiding the discussion on end-of-life issues in advance, lack of recognition of dementia as a terminal illness, and the fee-for-service predominant design of the healthcare system, which we illustrated in our previous publications [5,11]. The remarkably higher utilisation and longer length of stay of hospital/ICU admissions in the ET group echoed findings from the UK [10] and highlighted the urgent need to identify and develop preventive interventions such as HHC for those at high risk of ET. Further details on the pattern of avoidable aggressive interventions or life-sustaining treatments associated with the increased use of ICU services should be explored in the Asian context.

### 4.2. Impact of HHC on Burdensome End-of-Life Transitions

Much research on burdensome transitions or acute healthcare utilisation at the end of life has focused on residents living in long-term care facilities [8,9,29]. Sleeman and Leniz et al. analysed an electronic health records database in a mental health trust in London and, further, found that people with dementia at home are at a higher risk of burdensome end-of-life transitions than those in care homes [7,10]. To our knowledge, our study is the first one that targets the unmet need of these high-risk people with dementia by investigating the impact of HHC intervention on both ET and LT defined by Gozalo et al. [9].

The percentage of ET and LT in the group who received HHC in our study were 34.5% and 31.2%, respectively. In comparison with previous studies, Mitchell et al. included only those people with advanced dementia who died within 12 months after admissions to either the HHC programme or nursing home care and found that the ratio of hospitalisation within the 90 days prior to their last minimum dataset assessment was 31.5% in the HHC group, which was lower than the ratio of 43.7% in the nursing home group [22]. Jennings et al. followed a longitudinal cohort with dementia receiving a nurse practitioner-led comprehensive community care programme and showed 38% of the decedents were hospitalised in the last six months of life, whereas 69% died with hospice care, and 66% died at home [21]. The aforementioned two studies did not specifically report data on LT.

In our study, the HHC intervention had an opposite effect on ET and LT in people with dementia. The slightly higher risk of ET in the HHC group compared to those who did not have HHC may have occurred because people who received HHC may have had more multi-morbidities, more complex care needs, or have been at increased risk of using acute healthcare services before the HHC service [30,31]. For example, disabled people who received HHC in Taiwan had a higher risk of hospitalisation or ER transition after HHC (OR = 1.6, 95 % CI 1.41–1.83 and OR = 1.16, 95 % CI: 1.04–1.30, respectively) compared to the non-HHC group, even though HHC reduced hospital admissions by 80% and ER visits by 65% for people in the HHC group within the year after HHC service [30]. Another explanation is that better care coordination in home-based services compared with the usual care may identify more cases with unmet needs, leading to an increase in admissions for elderly people [32,33].

This interesting phenomenon of divergence between HHC and end-of-life transitions in different periods prior to death (early or late) may reflect the results of Jennings’ research [21]. This study showed that people with dementia who received the HHC programme and engaged in completion of the Physician Orders for Life-Sustaining Treatment (POLST) were more likely to be hospitalised in the last six months of life (43% versus 31%; *p* = 0.04) than those who received HHC but did not complete the POLST; however, the former group were also more likely to have had a discussion about hospice care (78% versus 64%; *p* = 0.01), die with hospice care (74% versus 62%; *p* = 0.03), and die at home (70% versus 59%; *p* = 0.04).

Our key finding is that the characteristics of HHC impacts on burdensome end-of-life transitions for people with dementia. HHC with longer duration, higher frequency, or that was provided continuously closer to the time of death may be factors indicating a better continuity of care (CoC) [34], and CoC is related to fewer hospitalisations and ER visits for people with dementia in the US Medicare system and a non-HHC population in Taiwan [35,36]. Continuous HHC may enhance the communication and relationship between patients, family members, and medical professionals and facilitate the ACP of substitute decision-makers and decision-making regarding do-not-resuscitation and do-not-hospitalise [21,37,38]. The obligated 24-h telephone support for clients in the Taiwanese HHC programme, which offers basic triage and care advice, may also contribute to the reduction of transitions [39].

### 4.3. Integration of Various Home-Based Services for Better End-of-Life Care

Although HBPLC showed increased odds of home death and had a heterogeneous effect on reduced hospitalisations at the end of life for people with different diseases [40,41], the evidence among people with dementia is unclear [16]. A recent Japanese cross-sectional nationwide death certificate study found that the density of primary care physicians in a region had a much stronger association with increased odds of home/nursing home death than the density of HBPLC did for people in chronic conditions, including dementia [42]. In our study, we found that HHC provided by both physicians and nurses was associated with a decreased risk of LT in contrast with nurse-only HHC. This implies that interdisciplinary coordination, which is also a core feature in HBPLC, leads to better outcomes and may reduce hospitalisation when death is approaching [43].

Considering the low coverage and late referral to specialist palliative care in people with dementia [11,14,42], especially for those living at home or in a care home, HHC should be embedded within a needs-orientated palliative care framework. HHC complements earlier delivery, broader interdisciplinary practice, better referral, or coordination with specialist palliative care to build up a seamless, integrated care model for supporting people in the long disease trajectory of dementia [44]. It is also important for health policymakers to design a value-based payment scheme to make this home-based integrated and continuous care model possible. To better understand this, the authors initiated the “Home-based Longitudinal Investigation of the Multidisciplinary Team Integrated Care (HOLISTIC)” in Taiwan [45], collecting data on a cohort served by an innovative “Integrated HHC programme” launched in 2016, which may provide more solid evidence in the future.

Our study has several limitations. With the fundamental weaknesses of an administrative database, we were unable to obtain information about the severity of cognitive impairment or comorbidities, lifestyle and social support factors, or functional status, which may lead to confounding. We were unable to define the place of residence of participants, which means an analysis of transitions between home and care home and the influence of place of care is not possible. A lack of detailed clinical information on the reasons for acute healthcare utilisation made the judgment of avoidable admissions or transitions difficult. There were no records of patients’ preferences and advance directives, nor the process of surrogate decision-making. It may be difficult to compare the results to other countries due to differences in healthcare delivery, reimbursement, or resources allocation. Finally, using old claims data for this study could lead to an incomplete depiction of the current situation. Future research will include more updated datasets. Despite these limitations, the association between routine HHC and the risk of end-of-life transition from a 12-year nationwide population-based cohort in Taiwan is novel and provides a key step for further investigation.

## 5. Conclusions

For people with dementia, HHC has contrasting effects on early and late burdensome end-of-life transitions prior to death. Characteristics of HHC such as intensity, continuity, or interdisciplinary coordination may reduce the risk of transitions at the end of life. We suggest that researchers and policymakers explore the synergy between routine HHC and specialist palliative care, and how they interact, to impact on acute healthcare utilisation in longitudinal studies through the trajectory from dementia diagnosis to death.

## Figures and Tables

**Table 1:** Characteristics of people with dementia and matched groups with or without burdensome end-of-life transition.

	All Decedents N = 61,399	Decedents Included in the Nested Case-Control Analysis			
		No Burdensome End-of-Life Transitions (Matched Controls) N = 34,910	Burdensome End-of-Life Transitions (Matched Cases) N = 17,455		
			Early Transition Only (Multiple Transitions Last 90 Days) N = 7891	Late Transition Only (Any Transition Last 3 Days) N = 8752	Both Early and Late Transitions N = 812
**Age when death, mean (SD)**	83.2 (9.7)	83.5 (9.6)	82.3 (9.6)	82.7 (10.1)	82.2 (10.2)
**Gender (female), ** ***n*** ** (%)**	29,595 (48.2)	17,099 (49.0)	3266 (41.4)	4486 (51.2)	339 (41.8)
***Characteristics at time of dementia diagnosis***					
**SES—Income ** ^**#**^ **, ** ***n*** ** (%) ** **Dependent ** **Fair ** **High ** **Very high**	11,934 (19.4) 32,609 (53.1) 16,076 (26.2) 780 (1.3)	6968 (20.0) 18,575 (53.2) 8955 (25.7) 412 (1.2)	1307 (16.6) 4660 (59.1) 1828 (23.2) 96 (1.2)	1776 (20.3) 4212 (48.1) 2621 (30.0) 143 (1.6)	143 (17.6) 426 (52.5) 239 (29.4) 4 (0.5)
**SES—Urbanisation, ** ***n*** ** (%) ** **1 most urbanised ** **2 ** **3 ** **4 most ruralised**	(Missing, *n* = 25,381; 41.3%) 15,745 (43.7) 15,494 (43.0) 4097 (11.4) 682 (1.9)	(Missing, *n* = 14,722; 42.2%) 8806 (43.6) 8733 (43.3) 2281 (11.3) 368 (1.8)	(Missing, *n* = 3153; 40.0%) 2005 (42.3) 2060 (43.5) 580 (12.2) 93 (2.0)	(Missing, *n* = 3580; 40.9%) 2356 (45.6) 2170 (42.0) 538 (10.4) 108 (2.1)	(Missing, *n* = 316; 38.9%) 211 (42.5) 218 (44.0) 55 (11.1) 12 (2.4)
**Age-adjusted CCI, mean (SD)**	7.4 (2.8)	7.3 (2.8)	7.7 (2.9)	7.3 (2.8)	7.9 (3.0)
**Comorbidities**					
**Cancer, ** ***n*** ** (%)**	8520 (13.9)	4699 (13.5)	1323 (16.8)	1101 (12.6)	158 (19.5)
**Heart failure, ** ***n*** ** (%)**	10,806 (17.6)	5998 (17.2)	1484 (18.8)	1573 (18.0)	174 (21.4)
**COPD, ** ***n*** ** (%)**	21,466 (35.0)	12,033 (34.5)	3066 (38.9)	2875 (32.9)	339 (41.8)
**Liver cirrhosis/ chronic liver Disease, ** ***n*** ** (%)**	11,946 (19.5)	6552 (18.8)	1768 (22.4)	1634 (18.7)	177 (21.8)
**Renal failure, ** ***n*** ** (%)**	1565 (2.6)	886 (2.5)	230 (2.9)	203 (2.3)	23 (2.8)
**Hypertension, ** ***n*** ** (%)**	40,441 (65.9)	22,805 (65.3)	5266 (66.7)	5720 (65.4)	555 (68.4)
**Diabetes, ** ***n*** ** (%)**	18,953 (30.9)	10,413 (29.8)	2664 (33.8)	2775 (31.7)	259 (31.9)
**Coronary artery disease, ** ***n*** ** (%)**	22,871 (37.3)	12,755 (36.5)	3082 (39.1)	3265 (37.3)	330 (40.6)
**Hyperlipidaemia, ** ***n*** ** (%)**	13,578 (22.1)	7408 (21.2)	1647 (20.9)	2181 (24.9)	174 (21.4)
**Atrial fibrillation, ** ***n*** ** (%)**	3954 (6.4)	2192 (6.3)	559 (7.1)	581 (6.6)	56 (6.9)
**Depression, ** ***n*** ** (%)**	8015 (13.1)	4321 (12.4)	1019 (12.9)	1313 (15.0)	121 (14.9)
***Characteristics in the follow-up***					
**Time from cohort entry to death (days), mean (SD)**	1030.7 (872.8)	1038.7 (859.4)	841.0 (770.4)	1093.5 (898.8)	887.2 (816.6)
**HHC related factors**					
**HHC, ** ***n***** (%)**	17,046 (27.8)	9670 (27.7)	2197 (27.8)	2277 (26.0)	251 (30.9)
**HHC (total counts/person), mean (SD)**	22.2 (27.4)	22.8 (27.3)	16.8 (21.8)	23.8 (28.8)	17.0 (24.1)
**Duration of HHC (days), median (Q1–Q3)**	234 (57–648)	245 (61–654)	151 (28–496)	256 (59–728)	127 (15–499)
**Frequency of HHC (counts /year/person), mean (SD)**	9.8 (6.7)	10.1 (6.7)	8.4 (6.4)	10.0 (6.8)	8.3 (6.5)
**Resource utilisation group of first HHC, ** ***n*** ** (%) ** **1 ** **2 ** **3 ** **4**	(missing n = 20; 0.1%) 895 (5.3) 11,958 (70.2) 3888 (22.8) 285 (1.7)	(missing *n* = 10; 0.1%) 487 (5.0) 6766 (70.0) 2242 (23.2) 165 (1.7)	(missing *n* = 4; 0.2%) 118 (5.4) 1458 (66.5) 567 (25.9) 50 (2.3)	(missing *n* = 5; 0.2%) 147 (6.5) 1663 (73.2) 437 (19.2) 25 (1.1)	11 (4.4) 168 (66.9) 68 (27.1) 4 (1.6)
**Time from cohort entry to first HHC (days), median (Q1–Q3)**	520 (145–1179)	520 (147–1165)	455 (133–1020)	578 (151–1255)	488 (115–1068)
**Time from last HHC to death, (days), median (Q1–Q3)**	29 (15–67)	30 (17–69)	46 (22–89)	18 (9–32)	22 (9–52)

COPD = chronic obstructive pulmonary disease, HHC = home healthcare, SD = standard deviation, SES = socioeconomic status, Q1 = first quartile, Q3 = third quartile, and CCI = Charlson Comorbidity Index. ^#^ Insurance premium-related income.

**Table 2:** Subgroup analysis of burdensome end-of-life transition in people with dementia who received home healthcare stratified by the characteristics of home healthcare.

	Early Burdensome Transition (Multiple Transitions Last 90 Days of Life)				Late Burdensome Transition (Any Transition Last 3 Days of Life)			
	Cases (*n*)	Controls (*n*)	Adjusted OR	95 % CI	Cases (*n*)	Controls (*n*)	Adjusted OR	95 % CI
**RUG**								
**1**	105	229	(Reference)		147	258	(Reference)	
**2**	1278	3223	0.85	0.67–1.08	1663	3543	0.94	0.81–1.10
**3**	486	1106	0.96	0.74–1.24	437	1136	0.91	0.80–1.03
**4**	29	88	0.71	0.44–1.15	25	77	0.91	0.79–1.06
**HHC by professionals**								
**HHC by nurse only**	410	783	(Reference)		410	748	(Reference)	
**HHC by physician and nurse**	2034	3863	1.01	0.88–1.15	1862	4266	0.80	0.70–0.91
**Duration of HHC (days)**								
**≤30**	670	861	(Reference)		413	894	(Reference)	
**>30 and ≤120**	471	821	0.80	0.66–0.98	404	840	0.69	0.56–0.85
**>120 and ≤365**	528	1157	0.58	0.48–0.71	504	1208	0.56	0.46–0.69
**>365**	779	1812	0.50	0.42–0.60	956	2077	0.60	0.50–0.73
**Frequency of HHC (counts/year)**								
**≤4**	933	1263	(Reference)		636	1318	(Reference)	
**5–8**	435	899	0.66	0.57–0.76	407	899	0.94	0.81–1.10
**9–16**	744	1598	0.64	0.56–0.72	789	1801	0.91	0.80–1.03
**>16**	336	891	0.52	0.45–0.61	445	1001	0.91	0.79–1.06
**Time between the dementia diagnosis and receiving the first HHC (years)**								
**<1**	1084	2079	(Reference)		895	1943	(Reference)	
**1–2**	804	1478	1.03	0.92–1.15	697	1526	0.99	0.87–1.11
**3–4**	210	434	0.91	0.76–1.10	230	566	0.87	0.73–1.03
**>4**	350	660	1.00	0.86–1.17	455	984	0.99	0.86–1.14
**Time between receiving the last HHC and death (days)**								
**>90**	569	999	(Reference)		265	994	(Reference)	
**31–90**	944	1303	1.28	1.12–1.46	334	1534	0.82	0.68–0.98
**16–30**	484	1245	0.69	0.59–0.80	692	1384	1.88	1.59–2.21
**0–15**	451	1104	0.73	0.62–0.84	986	1107	3.35	2.85–3.93

CI = confidence interval, HHC = home healthcare, RUG = resource utilisation groups, and OR = odds ratio. In the regression model, the early burdensome transition represents those with early transition only and those with both early and late transition; the late burdensome transition represents those with late transition only. All the analyses were adjusted for age, gender, socioeconomic status, age-adjusted Charlson Comorbidity Index, and comorbidities.

## Appendix A

**Table A1:** List of International Classification of Diseases, Ninth Revision, Clinical Modification (ICD-9-CM) code of the diseases.

Disease	ICD-9-CM code
Dementia	290.1x–209.4x, 291.2, 292.82, 294.1x, 294.8, 331.0, 331.1x, 331.2, 331.82
Stroke	430–438
Cancer	140–208
Heart Failure	428
Chronic obstructive pulmonary disease	491, 492, 494, 496
Liver cirrhosis/ chronic liver disease	571
Renal failure	585.4, 585.5, 585.6, 586
Hypertension	401, 402, 403, 404, 405
Diabetes	250, 251
Coronary artery disease	410–414
Hyperlipidaemia	272
Atrial fibrillation	427.31
Depression	296.2–296.36, 300.4, 311

**Table A2:** List of administrative claim codes for home healthcare in the National Health Insurance Research Database (NHIRD).

Code in NHIRD	Definition
A. In common area	
05307C	Fee for physician visit (per time)—for the first to fourth patient visited by one physician on the same day
05309C	Fee for physician visit (per time)—for the fifth to eighth patient visited by one physician on the same day
05301C	Fee for nurse visit (per time)—RUG 1, within reasonable caseload
05328C	Fee for nurse visit (per time)—RUG 1, beyond reasonable caseload
05303C	Fee for nurse visit (per time)—RUG 2, within reasonable caseload
05330C	Fee for nurse visit (per time)—RUG 2, beyond reasonable caseload
05305C	Fee for nurse visit (per time)—RUG 3, within reasonable caseload
05332C	Fee for nurse visit (per time)—RUG 3, beyond reasonable caseload
05321C	Fee for nurse visit (per time)—RUG 4, within reasonable caseload
05334C	Fee for nurse visit (per time)—RUG 4, beyond reasonable caseload
B. In specific rural area	
05308C	Fee for physician visit (per time)—for the first to fourth patient visited by one physician on the same day
05310C	Fee for physician visit (per time)—for the fifth to eighth patient visited by one physician on the same day
05302C	Fee for nurse visit (per time)—RUG 1, within reasonable caseload
05329C	Fee for nurse visit (per time)—RUG 1, beyond reasonable caseload
05304C	Fee for nurse visit (per time)—RUG 2, within reasonable caseload
05331C	Fee for nurse visit (per time)—RUG 2, beyond reasonable caseload
05306C	Fee for nurse visit (per time)—RUG 3, within reasonable caseload
05333C	Fee for nurse visit (per time)—RUG 3, beyond reasonable caseload
05322C	Fee for nurse visit (per time)—RUG 4, within reasonable caseload
05335C	Fee for nurse visit (per time)—RUG 4, beyond reasonable caseload

RUG = Classification of resource utilization groups.

**Table A3:** Numbers of cases with burdensome transitions at the end-of-lie and the controls in people with dementia who received home healthcare compared with those who had no home healthcare services.

	Early Burdensome Transition		Late Burdensome Transition	
	Cases	Controls	Cases	Controls
**Primary analysis**				
No HHC	6255 (71.87)	12755 (73.28)	6475 (73.98)	12,485 (71.33)
HHC	2448 (28.13)	4651 (26.72)	2277 (26.02)	5019 (28.67)
**Stratify by age when cohort entry**				
**40–64**				
No HHC	477 (81.12)	899 (85.05)	584 (89.02)	893 (82.08)
HHC	111 (18.88)	158 (14.95)	72 (10.98)	195 (17.92)
**65–84**				
No HHC	3715 (71.77)	7296 (72.80)	3799 (73.68)	7007 (70.74)
HHC	1461 (28.23)	2726 (27.20)	1357 (26.32)	2898 (29.26)
**>85**				
No HHC	2063 (71.19)	4560 (72.07)	2092 (71.16)	4585 (70.42)
HHC	876 (29.81)	1767 (27.93)	848 (28.84)	1926 (29.58)
**Stratify by time from dementia diagnosis to death (years)**				
**<1**				
No HHC	2642 (83.98)	4337 (84.71)	1900 (85.55)	3674 (84.44)
HHC	504 (16.02)	783 (15.29)	321 (14.45)	677 (15.56)
**1 to 2**				
No HHC	1983 (68.71)	4377 (72.17)	2198 (74.91)	4186 (72.93)
HHC	903 (31.29)	1688 (27.83)	736 (25.09)	1554 (27.07)
**3–5**				
No HHC	982 (62.59)	2362 (68.31)	1259 (68.65)	2404 (64.42)
HHC	587 (37.41)	1096 (31.69)	575 (31.35)	1328 (35.58)
**>5**				
No HHC	648 (58.80)	1679 (60.77)	1118 (63.41)	2221 (60.34)
HHC	454 (41.20)	1084 (39.23)	645 (36.59)	1460 (39.66)
**Stratify by year of death**				
**2002–2005**				
No HHC	1196 (82.71)	2400 (82.99)	560 (83.71)	1099 (82.14)
HHC	250 (17.29)	492 (17.01)	109 (16.29)	239 (17.86)
**2006–2009**				
No HHC	2628 (72.20)	5386 (73.98)	2043 (76.40)	3913 (73.17)
HHC	1012 (27.80)	1894 (26.02)	631 (23.60)	1435 (26.83)
**2010–2013**				
No HHC	2431 (67.21)	4969 (68.69)	3872 (71.58)	7473 (69.08)
HHC	1186 (32.79)	2265 (31.31)	1537 (28.42)	3345 (30.92)
**Stratify by stroke happened after cohort entry**				
**Yes**				
No HHC	2007 (56.73)	3632 (56.72)	1631 (56.67)	3715 (55.50)
HHC	1531 (43.27)	2771 (43.28)	1247 (43.33)	2979 (44.50)
**No**				
No HHC	4248 (82.25)	9123 (82.91)	4844 (82.47)	8770 (81.13)
HHC	917 (17.75)	1880 (17.09)	1030 (17.53)	2040 (18.87)

HHC = home healthcare and ICU = Intensive care unit.

**Table A4:** Sensitivity test for the influence by the missing data in the urbanisation variable on the risk of burdensome transition at the end-of-lie in people with dementia who received home healthcare compared with those who had no home healthcare services.

	Early Burdensome Transition		Late Burdensome Transition	
	Adjusted OR	95 % CI	Adjusted OR	95 % CI
**All people in the matched cases and controls**				
No HHC	1	(Reference)	1	(Reference)
HHC	1.14	1.08–1.21	0.89	0.83–0.94
**After deleting the people with missing data in urbanisation variable**				
No HHC	1	(Reference)	1	(Reference)
HHC	1.10	1.01–1.19	0.87	0.80–0.94

CI = confidence interval, HHC = home healthcare, and OR = odds ratio. In the regression model, the early burdensome transition represents those with early transition only and those with both early and late transition; the late burdensome transition represents those with late transition only. All the analyses were adjusted for age, gender, socioeconomic status, age-adjusted Charlson Comorbidity Index, and comorbidities.
